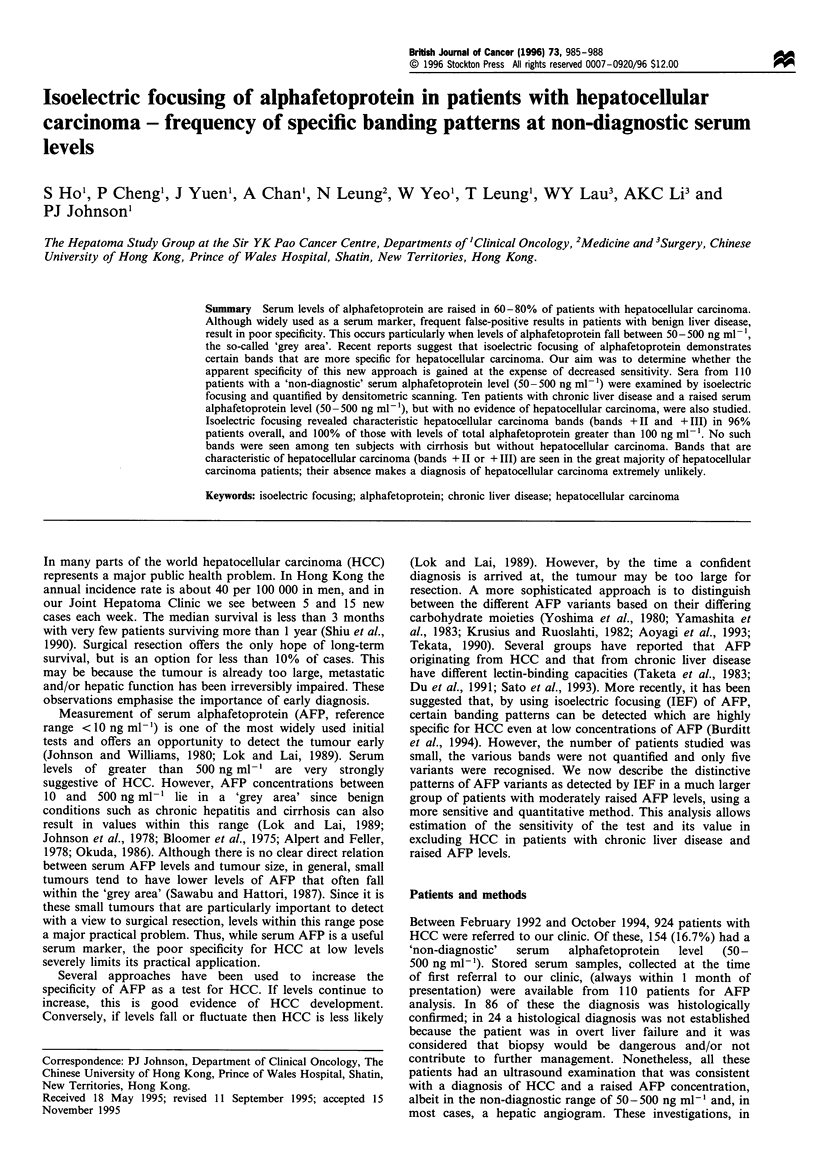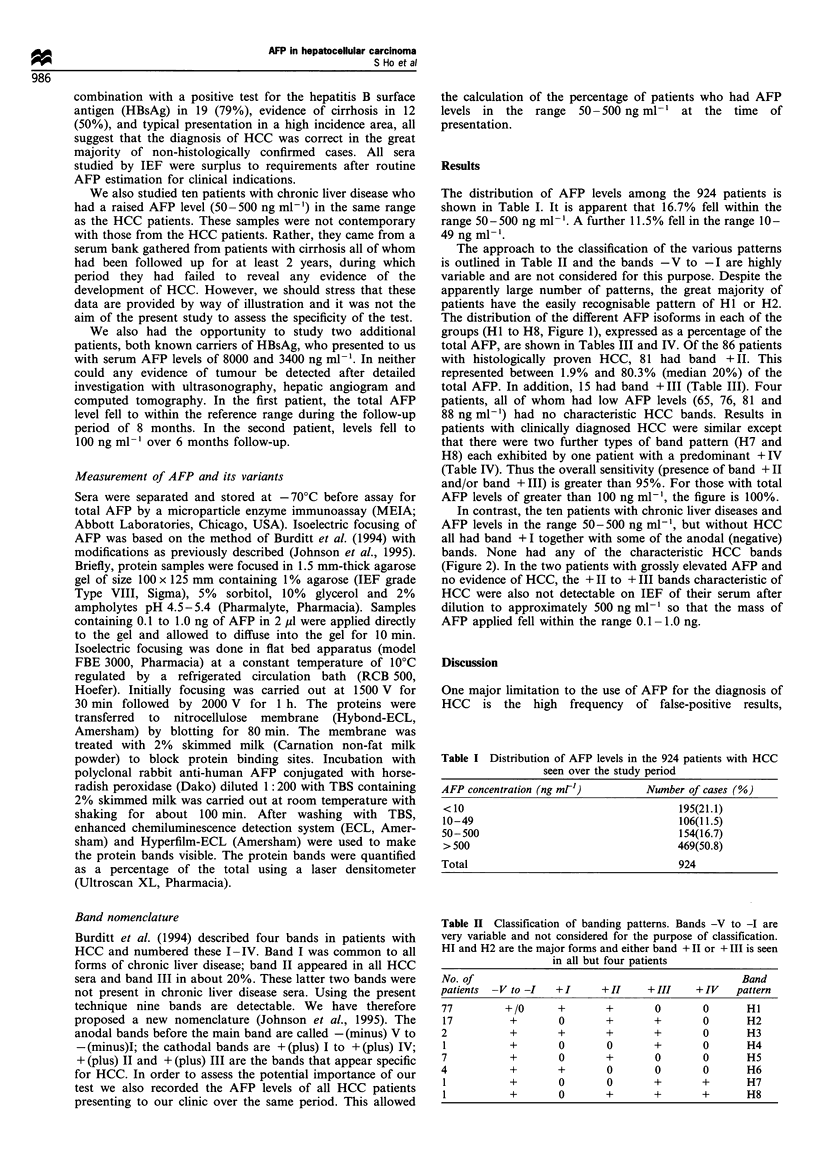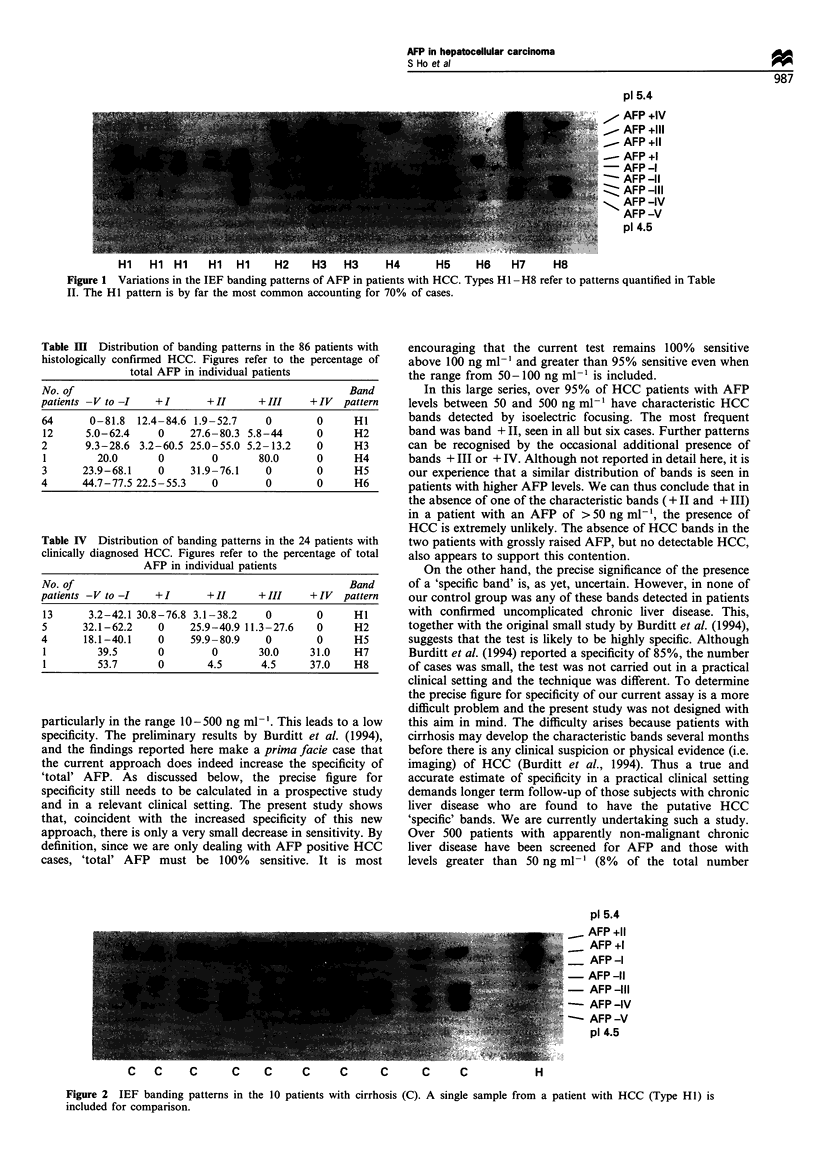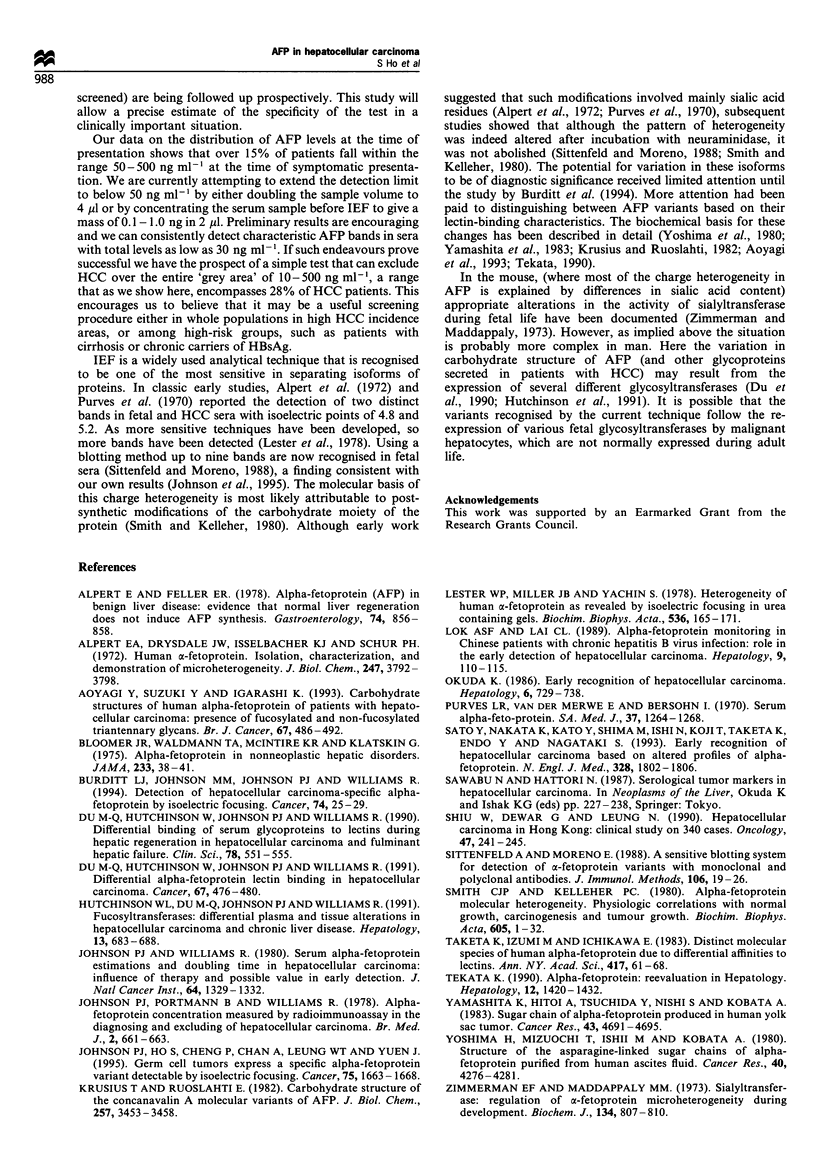# Isoelectric focusing of alphafetoprotein in patients with hepatocellular carcinoma--frequency of specific banding patterns at non-diagnostic serum levels.

**DOI:** 10.1038/bjc.1996.192

**Published:** 1996-04

**Authors:** S. Ho, P. Cheng, J. Yuen, A. Chan, N. Leung, W. Yeo, T. Leung, W. Y. Lau, A. K. Li, P. J. Johnson

**Affiliations:** Department of Clinical Oncology, Chinese University of Hong Kong, Prince of Wales Hospital, Shatin, New Territories.

## Abstract

**Images:**


British Journal of Cancer (1996) 73, 985-988
© 1996 Stockton Press All rights reserved 0007-0920/96 $12.00

### Isoelectric focusing of alphafetoprotein in patients with hepatocellular carcinoma – frequency of specific banding patterns at non-diagnostic serum levels

S Ho[1], P Cheng[1], J Yuen[1], A Chan[1], N Leung[2], W Yeo[1], T Leung[1], WY Lau[3], AKC Li[3] and PJ Johnson[1]

*The Hepatoma Study Group at the Sir YK Pao Cancer Centre, Departments of [1]Clinical Oncology, [2]Medicine and [3]Surgery, Chinese University of Hong Kong, Prince of Wales Hospital, Shatin, New Territories, Hong Kong.*

**Summary** Serum levels of alphafetoprotein are raised in 60–80% of patients with hepatocellular carcinoma. Although widely used as a serum marker, frequent false-positive results in patients with benign liver disease, result in poor specificity. This occurs particularly when levels of alphafetoprotein fall between 50–500 ng $ml^{-1}$, the so-called 'grey area'. Recent reports suggest that isoelectric focusing of alphafetoprotein demonstrates certain bands that are more specific for hepatocellular carcinoma. Our aim was to determine whether the apparent specificity of this new approach is gained at the expense of decreased sensitivity. Sera from 110 patients with a 'non-diagnostic' serum alphafetoprotein level (50–500 ng $ml^{-1}$) were examined by isoelectric focusing and quantified by densitometric scanning. Ten patients with chronic liver disease and a raised serum alphafetoprotein level (50–500 ng $ml^{-1}$), but with no evidence of hepatocellular carcinoma, were also studied. Isoelectric focusing revealed characteristic hepatocellular carcinoma bands (bands +II and +III) in 96% patients overall, and 100% of those with levels of total alphafetoprotein greater than 100 ng $ml^{-1}$. No such bands were seen among ten subjects with cirrhosis but without hepatocellular carcinoma. Bands that are characteristic of hepatocellular carcinoma (bands +II or +III) are seen in the great majority of hepatocellular carcinoma patients; their absence makes a diagnosis of hepatocellular carcinoma extremely unlikely.

**Keywords:** isoelectric focusing; alphafetoprotein; chronic liver disease; hepatocellular carcinoma

In many parts of the world hepatocellular carcinoma (HCC) represents a major public health problem. In Hong Kong the annual incidence rate is about 40 per 100 000 in men, and in our Joint Hepatoma Clinic we see between 5 and 15 new cases each week. The median survival is less than 3 months with very few patients surviving more than 1 year (Shiu *et al.*, 1990). Surgical resection offers the only hope of long-term survival, but is an option for less than 10% of cases. This may be because the tumour is already too large, metastatic and/or hepatic function has been irreversibly impaired. These observations emphasise the importance of early diagnosis.

Measurement of serum alphafetoprotein (AFP, reference range <10 ng $ml^{-1}$) is one of the most widely used initial tests and offers an opportunity to detect the tumour early (Johnson and Williams, 1980; Lok and Lai, 1989). Serum levels of greater than 500 ng $ml^{-1}$ are very strongly suggestive of HCC. However, AFP concentrations between 10 and 500 ng $ml^{-1}$ lie in a 'grey area' since benign conditions such as chronic hepatitis and cirrhosis can also result in values within this range (Lok and Lai, 1989; Johnson *et al.*, 1978; Bloomer *et al.*, 1975; Alpert and Feller, 1978; Okuda, 1986). Although there is no clear direct relation between serum AFP levels and tumour size, in general, small tumours tend to have lower levels of AFP that often fall within the 'grey area' (Sawabu and Hattori, 1987). Since it is these small tumours that are particularly important to detect with a view to surgical resection, levels within this range pose a major practical problem. Thus, while serum AFP is a useful serum marker, the poor specificity for HCC at low levels severely limits its practical application.

Several approaches have been used to increase the specificity of AFP as a test for HCC. If levels continue to increase, this is good evidence of HCC development. Conversely, if levels fall or fluctuate then HCC is less likely (Lok and Lai, 1989). However, by the time a confident diagnosis is arrived at, the tumour may be too large for resection. A more sophisticated approach is to distinguish between the different AFP variants based on their differing carbohydrate moieties (Yoshima *et al.*, 1980; Yamashita *et al.*, 1983; Krusius and Ruoslahti, 1982; Aoyagi *et al.*, 1993; Tekata, 1990). Several groups have reported that AFP originating from HCC and that from chronic liver disease have different lectin-binding capacities (Taketa *et al.*, 1983; Du *et al.*, 1991; Sato *et al.*, 1993). More recently, it has been suggested that, by using isoelectric focusing (IEF) of AFP, certain banding patterns can be detected which are highly specific for HCC even at low concentrations of AFP (Burditt *et al.*, 1994). However, the number of patients studied was small, the various bands were not quantified and only five variants were recognised. We now describe the distinctive patterns of AFP variants as detected by IEF in a much larger group of patients with moderately raised AFP levels, using a more sensitive and quantitative method. This analysis allows estimation of the sensitivity of the test and its value in excluding HCC in patients with chronic liver disease and raised AFP levels.

### Patients and methods

Between February 1992 and October 1994, 924 patients with HCC were referred to our clinic. Of these, 154 (16.7%) had a 'non-diagnostic' serum alphafetoprotein level (50–500 ng $ml^{-1}$). Stored serum samples, collected at the time of first referral to our clinic, (always within 1 month of presentation) were available from 110 patients for AFP analysis. In 86 of these the diagnosis was histologically confirmed; in 24 a histological diagnosis was not established because the patient was in overt liver failure and it was considered that biopsy would be dangerous and/or not contribute to further management. Nonetheless, all these patients had an ultrasound examination that was consistent with a diagnosis of HCC and a raised AFP concentration, albeit in the non-diagnostic range of 50–500 ng $ml^{-1}$ and, in most cases, a hepatic angiogram. These investigations, in

Correspondence: PJ Johnson, Department of Clinical Oncology, The Chinese University of Hong Kong, Prince of Wales Hospital, Shatin, New Territories, Hong Kong.
Received 18 May 1995; revised 11 September 1995; accepted 15 November 1995

combination with a positive test for the hepatitis B surface antigen (HBsAg) in 19 (79%), evidence of cirrhosis in 12 (50%), and typical presentation in a high incidence area, all suggest that the diagnosis of HCC was correct in the great majority of non-histologically confirmed cases. All sera studied by IEF were surplus to requirements after routine AFP estimation for clinical indications.

We also studied ten patients with chronic liver disease who had a raised AFP level (50–500 ng $ml^{-1}$) in the same range as the HCC patients. These samples were not contemporary with those from the HCC patients. Rather, they came from a serum bank gathered from patients with cirrhosis all of whom had been followed up for at least 2 years, during which period they had failed to reveal any evidence of the development of HCC. However, we should stress that these data are provided by way of illustration and it was not the aim of the present study to assess the specificity of the test.

We also had the opportunity to study two additional patients, both known carriers of HBsAg, who presented to us with serum AFP levels of 8000 and 3400 ng $ml^{-1}$. In neither could any evidence of tumour be detected after detailed investigation with ultrasonography, hepatic angiogram and computed tomography. In the first patient, the total AFP level fell to within the reference range during the follow-up period of 8 months. In the second patient, levels fell to 100 ng $ml^{-1}$ over 6 months follow-up.

### *Measurement of AFP and its variants*

Sera were separated and stored at −70°C before assay for total AFP by a microparticle enzyme immunoassay (MEIA; Abbott Laboratories, Chicago, USA). Isoelectric focusing of AFP was based on the method of Burditt *et al.* (1994) with modifications as previously described (Johnson *et al.*, 1995). Briefly, protein samples were focused in 1.5 mm-thick agarose gel of size 100 × 125 mm containing 1% agarose (IEF grade Type VIII, Sigma), 5% sorbitol, 10% glycerol and 2% ampholytes pH 4.5–5.4 (Pharmalyte, Pharmacia). Samples containing 0.1 to 1.0 ng of AFP in 2 μl were applied directly to the gel and allowed to diffuse into the gel for 10 min. Isoelectric focusing was done in flat bed apparatus (model FBE 3000, Pharmacia) at a constant temperature of 10°C regulated by a refrigerated circulation bath (RCB 500, Hoefer). Initially focusing was carried out at 1500 V for 30 min followed by 2000 V for 1 h. The proteins were transferred to nitrocellulose membrane (Hybond-ECL, Amersham) by blotting for 80 min. The membrane was treated with 2% skimmed milk (Carnation non-fat milk powder) to block protein binding sites. Incubation with polyclonal rabbit anti-human AFP conjugated with horseradish peroxidase (Dako) diluted 1:200 with TBS containing 2% skimmed milk was carried out at room temperature with shaking for about 100 min. After washing with TBS, enhanced chemiluminescence detection system (ECL, Amersham) and Hyperfilm-ECL (Amersham) were used to make the protein bands visible. The protein bands were quantified as a percentage of the total using a laser densitometer (Ultroscan XL, Pharmacia).

### *Band nomenclature*

Burditt *et al.* (1994) described four bands in patients with HCC and numbered these I–IV. Band I was common to all forms of chronic liver disease; band II appeared in all HCC sera and band III in about 20%. These latter two bands were not present in chronic liver disease sera. Using the present technique nine bands are detectable. We have therefore proposed a new nomenclature (Johnson *et al.*, 1995). The anodal bands before the main band are called −(minus) V to −(minus)I; the cathodal bands are +(plus) I to +(plus) IV; +(plus) II and +(plus) III are the bands that appear specific for HCC. In order to assess the potential importance of our test we also recorded the AFP levels of all HCC patients presenting to our clinic over the same period. This allowed the calculation of the percentage of patients who had AFP levels in the range 50–500 ng $ml^{-1}$ at the time of presentation.

### Results

The distribution of AFP levels among the 924 patients is shown in Table I. It is apparent that 16.7% fell within the range 50–500 ng $ml^{-1}$. A further 11.5% fell in the range 10–49 ng $ml^{-1}$.

The approach to the classification of the various patterns is outlined in Table II and the bands −V to −I are highly variable and are not considered for this purpose. Despite the apparently large number of patterns, the great majority of patients have the easily recognisable pattern of H1 or H2. The distribution of the different AFP isoforms in each of the groups (H1 to H8, Figure 1), expressed as a percentage of the total AFP, are shown in Tables III and IV. Of the 86 patients with histologically proven HCC, 81 had band +II. This represented between 1.9% and 80.3% (median 20%) of the total AFP. In addition, 15 had band +III (Table III). Four patients, all of whom had low AFP levels (65, 76, 81 and 88 ng $ml^{-1}$) had no characteristic HCC bands. Results in patients with clinically diagnosed HCC were similar except that there were two further types of band pattern (H7 and H8) each exhibited by one patient with a predominant +IV (Table IV). Thus the overall sensitivity (presence of band +II and/or band +III) is greater than 95%. For those with total AFP levels of greater than 100 ng $ml^{-1}$, the figure is 100%.

In contrast, the ten patients with chronic liver diseases and AFP levels in the range 50–500 ng $ml^{-1}$, but without HCC all had band +I together with some of the anodal (negative) bands. None had any of the characteristic HCC bands (Figure 2). In the two patients with grossly elevated AFP and no evidence of HCC, the +II to +III bands characteristic of HCC were also not detectable on IEF of their serum after dilution to approximately 500 ng $ml^{-1}$ so that the mass of AFP applied fell within the range 0.1–1.0 ng.

### Discussion

One major limitation to the use of AFP for the diagnosis of HCC is the high frequency of false-positive results,

**Table I** Distribution of AFP levels in the 924 patients with HCC seen over the study period

| *AFP concentration (ng $ml^{-1}$)* | *Number of cases (%)* |
|---|---|
| <10 | 195(21.1) |
| 10–49 | 106(11.5) |
| 50–500 | 154(16.7) |
| >500 | 469(50.8) |
| Total | 924 |

**Table II** Classification of banding patterns. Bands −V to −I are very variable and not considered for the purpose of classification. HI and H2 are the major forms and either band +II or +III is seen in all but four patients

| *No. of patients* | *−V to −I* | *+I* | *+II* | *+III* | *+IV* | *Band pattern* |
|---|---|---|---|---|---|---|
| 77 | +/0 | + | + | 0 | 0 | H1 |
| 17 | + | 0 | + | + | 0 | H2 |
| 2 | + | + | + | + | 0 | H3 |
| 1 | + | 0 | 0 | + | 0 | H4 |
| 7 | + | 0 | + | 0 | 0 | H5 |
| 4 | + | + | 0 | 0 | 0 | H6 |
| 1 | + | 0 | 0 | + | + | H7 |
| 1 | + | 0 | + | + | + | H8 |

**Figure 1** Variations in the IEF banding patterns of AFP in patients with HCC. Types H1–H8 refer to patterns quantified in Table II. The H1 pattern is by far the most common accounting for 70% of cases.

**Table III** Distribution of banding patterns in the 86 patients with histologically confirmed HCC. Figures refer to the percentage of total AFP in individual patients

| *No. of patients* | *−V to −I* | *+I* | *+II* | *+III* | *+IV* | *Band pattern* |
|---|---|---|---|---|---|---|
| 64 | 0–81.8 | 12.4–84.6 | 1.9–52.7 | 0 | 0 | H1 |
| 12 | 5.0–62.4 | 0 | 27.6–80.3 | 5.8–44 | 0 | H2 |
| 2 | 9.3–28.6 | 3.2–60.5 | 25.0–55.0 | 5.2–13.2 | 0 | H3 |
| 1 | 20.0 | 0 | 0 | 80.0 | 0 | H4 |
| 3 | 23.9–68.1 | 0 | 31.9–76.1 | 0 | 0 | H5 |
| 4 | 44.7–77.5 | 22.5–55.3 | 0 | 0 | 0 | H6 |

**Table IV** Distribution of banding patterns in the 24 patients with clinically diagnosed HCC. Figures refer to the percentage of total AFP in individual patients

| *No. of patients* | *−V to −I* | *+I* | *+II* | *+III* | *+IV* | *Band pattern* |
|---|---|---|---|---|---|---|
| 13 | 3.2–42.1 | 30.8–76.8 | 3.1–38.2 | 0 | 0 | H1 |
| 5 | 32.1–62.2 | 0 | 25.9–40.9 | 11.3–27.6 | 0 | H2 |
| 4 | 18.1–40.1 | 0 | 59.9–80.9 | 0 | 0 | H5 |
| 1 | 39.5 | 0 | 0 | 30.0 | 31.0 | H7 |
| 1 | 53.7 | 0 | 4.5 | 4.5 | 37.0 | H8 |

particularly in the range 10–500 ng $ml^{-1}$. This leads to a low specificity. The preliminary results by Burditt *et al.* (1994), and the findings reported here make a *prima facie* case that the current approach does indeed increase the specificity of 'total' AFP. As discussed below, the precise figure for specificity still needs to be calculated in a prospective study and in a relevant clinical setting. The present study shows that, coincident with the increased specificity of this new approach, there is only a very small decrease in sensitivity. By definition, since we are only dealing with AFP positive HCC cases, 'total' AFP must be 100% sensitive. It is most encouraging that the current test remains 100% sensitive above 100 ng $ml^{-1}$ and greater than 95% sensitive even when the range from 50–100 ng $ml^{-1}$ is included.

In this large series, over 95% of HCC patients with AFP levels between 50 and 500 ng $ml^{-1}$ have characteristic HCC bands detected by isoelectric focusing. The most frequent band was band +II, seen in all but six cases. Further patterns can be recognised by the occasional additional presence of bands +III or +IV. Although not reported in detail here, it is our experience that a similar distribution of bands is seen in patients with higher AFP levels. We can thus conclude that in the absence of one of the characteristic bands (+II and +III) in a patient with an AFP of >50 ng $ml^{-1}$, the presence of HCC is extremely unlikely. The absence of HCC bands in the two patients with grossly raised AFP, but no detectable HCC, also appears to support this contention.

On the other hand, the precise significance of the presence of a 'specific band' is, as yet, uncertain. However, in none of our control group was any of these bands detected in patients with confirmed uncomplicated chronic liver disease. This, together with the original small study by Burditt *et al.* (1994), suggests that the test is likely to be highly specific. Although Burditt *et al.* (1994) reported a specificity of 85%, the number of cases was small, the test was not carried out in a practical clinical setting and the technique was different. To determine the precise figure for specificity of our current assay is a more difficult problem and the present study was not designed with this aim in mind. The difficulty arises because patients with cirrhosis may develop the characteristic bands several months before there is any clinical suspicion or physical evidence (i.e. imaging) of HCC (Burditt *et al.*, 1994). Thus a true and accurate estimate of specificity in a practical clinical setting demands longer term follow-up of those subjects with chronic liver disease who are found to have the putative HCC 'specific' bands. We are currently undertaking such a study. Over 500 patients with apparently non-malignant chronic liver disease have been screened for AFP and those with levels greater than 50 ng $ml^{-1}$ (8% of the total number

**Figure 2** IEF banding patterns in the 10 patients with cirrhosis (C). A single sample from a patient with HCC (Type H1) is included for comparison.

screened) are being followed up prospectively. This study will allow a precise estimate of the specificity of the test in a clinically important situation.

Our data on the distribution of AFP levels at the time of presentation shows that over 15% of patients fall within the range 50–500 ng $ml^{-1}$ at the time of symptomatic presentation. We are currently attempting to extend the detection limit to below 50 ng $ml^{-1}$ by either doubling the sample volume to 4 $\mu l$ or by concentrating the serum sample before IEF to give a mass of 0.1–1.0 ng in 2 $\mu l$. Preliminary results are encouraging and we can consistently detect characteristic AFP bands in sera with total levels as low as 30 ng $ml^{-1}$. If such endeavours prove successful we have the prospect of a simple test that can exclude HCC over the entire 'grey area' of 10–500 ng $ml^{-1}$, a range that as we show here, encompasses 28% of HCC patients. This encourages us to believe that it may be a useful screening procedure either in whole populations in high HCC incidence areas, or among high-risk groups, such as patients with cirrhosis or chronic carriers of HBsAg.

IEF is a widely used analytical technique that is recognised to be one of the most sensitive in separating isoforms of proteins. In classic early studies, Alpert *et al.* (1972) and Purves *et al.* (1970) reported the detection of two distinct bands in fetal and HCC sera with isoelectric points of 4.8 and 5.2. As more sensitive techniques have been developed, so more bands have been detected (Lester *et al.*, 1978). Using a blotting method up to nine bands are now recognised in fetal sera (Sittenfeld and Moreno, 1988), a finding consistent with our own results (Johnson *et al.*, 1995). The molecular basis of this charge heterogeneity is most likely attributable to post-synthetic modifications of the carbohydrate moiety of the protein (Smith and Kelleher, 1980). Although early work suggested that such modifications involved mainly sialic acid residues (Alpert *et al.*, 1972; Purves *et al.*, 1970), subsequent studies showed that although the pattern of heterogeneity was indeed altered after incubation with neuraminidase, it was not abolished (Sittenfeld and Moreno, 1988; Smith and Kelleher, 1980). The potential for variation in these isoforms to be of diagnostic significance received limited attention until the study by Burditt *et al.* (1994). More attention had been paid to distinguishing between AFP variants based on their lectin-binding characteristics. The biochemical basis for these changes has been described in detail (Yoshima *et al.*, 1980; Yamashita *et al.*, 1983; Krusius and Ruoslahti, 1982; Aoyagi *et al.*, 1993; Tekata, 1990).

In the mouse, (where most of the charge heterogeneity in AFP is explained by differences in sialic acid content) appropriate alterations in the activity of sialyltransferase during fetal life have been documented (Zimmerman and Maddappaly, 1973). However, as implied above the situation is probably more complex in man. Here the variation in carbohydrate structure of AFP (and other glycoproteins secreted in patients with HCC) may result from the expression of several different glycosyltransferases (Du *et al.*, 1990; Hutchinson *et al.*, 1991). It is possible that the variants recognised by the current technique follow the re-expression of various fetal glycosyltransferases by malignant hepatocytes, which are not normally expressed during adult life.

**Acknowledgements**
This work was supported by an Earmarked Grant from the Research Grants Council.

### References

ALPERT E AND FELLER ER. (1978). Alpha-fetoprotein (AFP) in benign liver disease: evidence that normal liver regeneration does not induce AFP synthesis. *Gastroenterology*, **74**, 856–858.

ALPERT EA, DRYSDALE JW, ISSELBACHER KJ AND SCHUR PH. (1972). Human α-fetoprotein. Isolation, characterization, and demonstration of microheterogeneity. *J. Biol. Chem.*, **247**, 3792–3798.

AOYAGI Y, SUZUKI Y AND IGARASHI K. (1993). Carbohydrate structures of human alpha-fetoprotein of patients with hepatocellular carcinoma: presence of fucosylated and non-fucosylated triantennary glycans. *Br. J. Cancer*, **67**, 486–492.

BLOOMER JR, WALDMANN TA, MCINTIRE KR AND KLATSKIN G. (1975). Alpha-fetoprotein in nonneoplastic hepatic disorders. *JAMA*, **233**, 38–41.

BURDITT LJ, JOHNSON MM, JOHNSON PJ AND WILLIAMS R. (1994). Detection of hepatocellular carcinoma-specific alpha-fetoprotein by isoelectric focusing. *Cancer*, **74**, 25–29.

DU M-Q, HUTCHINSON W, JOHNSON PJ AND WILLIAMS R. (1990). Differential binding of serum glycoproteins to lectins during hepatic regeneration in hepatocellular carcinoma and fulminant hepatic failure. *Clin. Sci.*, **78**, 551–555.

DU M-Q, HUTCHINSON W, JOHNSON PJ AND WILLIAMS R. (1991). Differential alpha-fetoprotein lectin binding in hepatocellular carcinoma. *Cancer*, **67**, 476–480.

HUTCHINSON WL, DU M-Q, JOHNSON PJ AND WILLIAMS R. (1991). Fucosyltransferases: differential plasma and tissue alterations in hepatocellular carcinoma and chronic liver disease. *Hepatology*, **13**, 683–688.

JOHNSON PJ AND WILLIAMS R. (1980). Serum alpha-fetoprotein estimations and doubling time in hepatocellular carcinoma: influence of therapy and possible value in early detection. *J. Natl Cancer Inst.*, **64**, 1329–1332.

JOHNSON PJ, PORTMANN B AND WILLIAMS R. (1978). Alpha-fetoprotein concentration measured by radioimmunoassay in the diagnosing and excluding of hepatocellular carcinoma. *Br. Med. J.*, **2**, 661–663.

JOHNSON PJ, HO S, CHENG P, CHAN A, LEUNG WT AND YUEN J. (1995). Germ cell tumors express a specific alpha-fetoprotein variant detectable by isoelectric focusing. *Cancer*, **75**, 1663–1668.

KRUSIUS T AND RUOSLAHTI E. (1982). Carbohydrate structure of the concanavalin A molecular variants of AFP. *J. Biol. Chem.*, **257**, 3453–3458.

LESTER WP, MILLER JB AND YACHIN S. (1978). Heterogeneity of human α-fetoprotein as revealed by isoelectric focusing in urea containing gels. *Biochim. Biophys. Acta.*, **536**, 165–171.

LOK ASF AND LAI CL. (1989). Alpha-fetoprotein monitoring in Chinese patients with chronic hepatitis B virus infection: role in the early detection of hepatocellular carcinoma. *Hepatology*, **9**, 110–115.

OKUDA K. (1986). Early recognition of hepatocellular carcinoma. *Hepatology*, **6**, 729–738.

PURVES LR, VAN DER MERWE E AND BERSOHN I. (1970). Serum alpha-feto-protein. *SA. Med. J.*, **37**, 1264–1268.

SATO Y, NAKATA K, KATO Y, SHIMA M, ISHI N, KOJI T, TAKETA K, ENDO Y AND NAGATAKI S. (1993). Early recognition of hepatocellular carcinoma based on altered profiles of alpha-fetoprotein. *N. Engl. J. Med.*, **328**, 1802–1806.

SAWABU N AND HATTORI N. (1987). Serological tumor markers in hepatocellular carcinoma. In *Neoplasms of the Liver*, Okuda K and Ishak KG (eds) pp. 227–238, Springer: Tokyo.

SHIU W, DEWAR G AND LEUNG N. (1990). Hepatocellular carcinoma in Hong Kong: clinical study on 340 cases. *Oncology*, **47**, 241–245.

SITTENFELD A AND MORENO E. (1988). A sensitive blotting system for detection of α-fetoprotein variants with monoclonal and polyclonal antibodies. *J. Immunol. Methods*, **106**, 19–26.

SMITH CJP AND KELLEHER PC. (1980). Alpha-fetoprotein molecular heterogeneity. Physiologic correlations with normal growth, carcinogenesis and tumour growth. *Biochim. Biophys. Acta*, **605**, 1–32.

TAKETA K, IZUMI M AND ICHIKAWA E. (1983). Distinct molecular species of human alpha-fetoprotein due to differential affinities to lectins. *Ann. NY. Acad. Sci.*, **417**, 61–68.

TEKATA K. (1990). Alpha-fetoprotein: reevaluation in Hepatology. *Hepatology*, **12**, 1420–1432.

YAMASHITA K, HITOI A, TSUCHIDA Y, NISHI S AND KOBATA A. (1983). Sugar chain of alpha-fetoprotein produced in human yolk sac tumor. *Cancer Res.*, **43**, 4691–4695.

YOSHIMA H, MIZUOCHI T, ISHII M AND KOBATA A. (1980). Structure of the asparagine-linked sugar chains of alpha-fetoprotein purified from human ascites fluid. *Cancer Res.*, **40**, 4276–4281.

ZIMMERMAN EF AND MADDAPPALY MM. (1973). Sialyltransferase: regulation of α-fetoprotein microheterogeneity during development. *Biochem. J.*, **134**, 807–810.